# Synthesis and crystal structure of a new magnesium phosphate Na_3_RbMg_7_(PO_4_)_6_

**DOI:** 10.1107/S2056989017006363

**Published:** 2017-05-05

**Authors:** Teycir Ben Hamed, Amal Boukhris, Abdessalem Badri, Mongi Ben Amara

**Affiliations:** a Unité de recherche, Matériaux Inorganiques, Faculté des Sciences, Université de Monastir, 5019, Monastir, Tunisia

**Keywords:** crystal structure, magnesium phosphate, anionic framework

## Abstract

A new magnesium phosphate, Na_3_RbMg_7_(PO_4_)_6_ [tris­odium rubidium hepta­magnesium hexakis(ortho­phosphate)], has been synthesized as single crystals by the flux method and exhibits a new structure type. Its original structure is built up from MgO_*x*_ (*x* = 5 and 6) polyhedra linked directly to each other through common corners or edges and reinforced by corner-sharing with PO_4_ tetra­hedra. The resulting anionic three-dimensional framework leads to the formation of channels along the [010] direction, in which the Na^+^ cations are located, while the Rb^+^ cations are located in large inter­stitial cavities.

## Abstract 2

Trisodium rubidium hepta­magnesium hexakis(ortho­phosphate) exhibits a new structure type, with MgO_*x*_ (*x* = 5 and 6) polyhedra linked directly to each other through common corners or edges and reinforced by corner-sharing with PO_4_ tetra­hedra. The resulting anionic three-dimensional framework leads to the formation of channels in which the Na^+^ cations are located, while the Rb^+^ cations are located in large inter­stitial cavities.

## Chemical context   

Magnesium phosphates are of increasing inter­est because of their potential applications as host materials for optically active rare earth ions (Seo, 2013 ▸; Kim *et al.*, 2013 ▸; Boukhris *et al.*, 2015 ▸). Moreover, these materials are very attractive in terms of basic research because they exhibit a rich structural chemistry due to their polymorphism (Ait Benhamou *et al.*, 2010 ▸; Orlova *et al.*, 2015 ▸).

Among the variety of magnesium monophosphates synthesized and characterized up to now, only four compounds belong to the system Na_3_PO_4_–Mg_3_(PO_4_)_2_, namely NaMgPO_4_, NaMg_4_(PO_4_)_3_, Na_2_Mg_5_(PO_4_)_4_ and Na_4_Mg(PO_4_)_2_ (Imura & Kawahara, 1997 ▸; Ben Amara *et al.*, 1983 ▸; Yamakawa *et al.*, 1994 ▸; Ghorbel *et al.*, 1974 ▸). NaMgPO_4_ compound crystallizes in the ortho­rhom­bic system with space group *P*2_1_2_1_2_1_. Its structure involves MgO_6_ and MgO_5_ polyhedra linked by the monophosphate groups that form a three-dimensional framework. NaMg_4_(PO_4_)_3_ is also ortho­rhom­bic, space group *Pnma*. Its structure is built up from three kinds of MgO_5_ units sharing edges and corners and linked to each other by the PO_4_ tetra­hedra, leading to a three-dimensional framework. Na_2_Mg_5_(PO_4_)_4_, synthesized under pressure, crystallizes in the triclinic system. Its structure results from a three-dimensional framework of MgO_6_ and MgO_5_ polyhedra connected either directly *via* common corners or by means of the phosphate groups. Na_4_Mg(PO_4_)_2_ exhibits two polymorphs, which were only identified by their powder diffraction patterns.

Starting from these compounds, suitable replacements of magnesium and/or sodium by large cations induces their transformation into several structural types for different Mg/P atomic ratios. Na*M*Mg(PO_4_)_2_ (*M* = Ca, Sr and Ba) compounds are related to the glaserite-type structure (Alkemper & Fuess, 1998 ▸; Boukhris *et al.*, 2012 ▸, 2013 ▸). They adopt an anionic two-dimensional network with different symmetries as a function of the size of the *M* ^2+^ cation. For an atomic ratio *M*:P of 7:6, magnesium phosphate compounds adopt a three-dimensional network related to the fillowite-type structure, as observed in Na_4_Ca_4_Mg_21_(PO_4_)_18_, Na_2_CaMg_7_(PO_4_)_6_ and Na_2.5_Y_0.5_Mg_7_(PO_4_)_6_ (Domanskii *et al.*, 1982 ▸; McCoy *et al.*, 1994 ▸; Jerbi *et al.*, 2010*a* ▸). All of them crystallize with trigonal symmetry (space group *R* 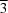) and differ only by their cationic distributions. Three-dimensional anionic networks includes also original structures such as those observed in Na_18_Ca_13_Mg_5_(PO_4_)_18_, NaCa_9_Mg(PO_4_)_7_, Na_7_ *Ln*Mg_13_(PO_4_)_12_ (*Ln* = La, Eu, Nd) (Yamakawa *et al.*, 1994 ▸; Morozov *et al.*, 1997 ▸; Jerbi *et al.*, 2010*b* ▸, 2012 ▸).

As a contribution to the investigation of the above-mentioned systems, we report here the structural characterization of a new magnesium phosphate Na_3_RbMg_7_(PO_4_)_6_, which is, to our knowledge, the first magnesium phosphate revealing an original structure for an atomic ratio Mg/P equal to 7/6.

## Structural commentary   

**Figure 1:**
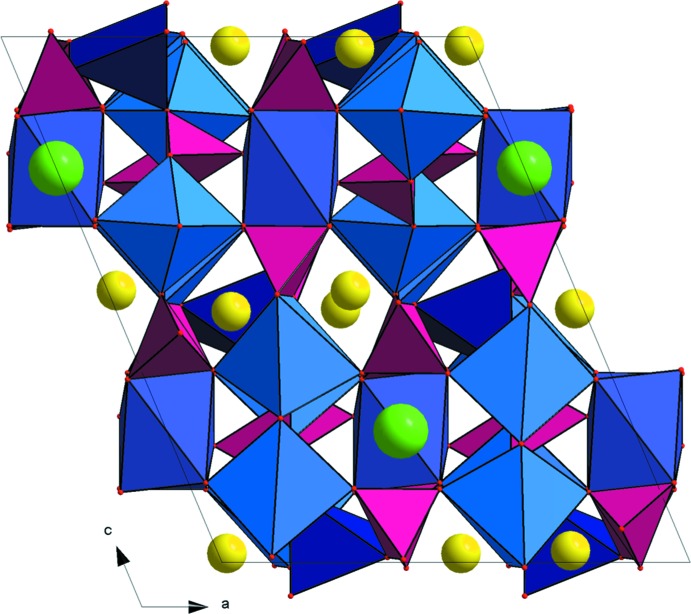
A view of the Na_3_RbMg_7_(PO_4_)_6_ structure along [010]. Colour key: MgO_*x*_ (*x* = 5 and 6; blue polyhedra), PO_4_ (purple polyhedra), Rb (green spheres) and Na (yellow spheres).

To the best of our knowledge, Na_3_RbMg_7_(PO_4_)_6_ exhibits a new structure type. A projection along the [010] direction of its structure (Fig. 1 ▸) clearly evidences the three-dimensional character of its anionic framework, which is built up from five different polyhedra MgO_*x*_ (*x* = 5, 6) and three kinds of PO_4_ tetra­hedra connected together by sharing edges and corners. The Na^+^ cations are located within channels running along the [010] direction while the Rb^+^ cations are found in the large inter­stitial cavities.

**Figure 2:**
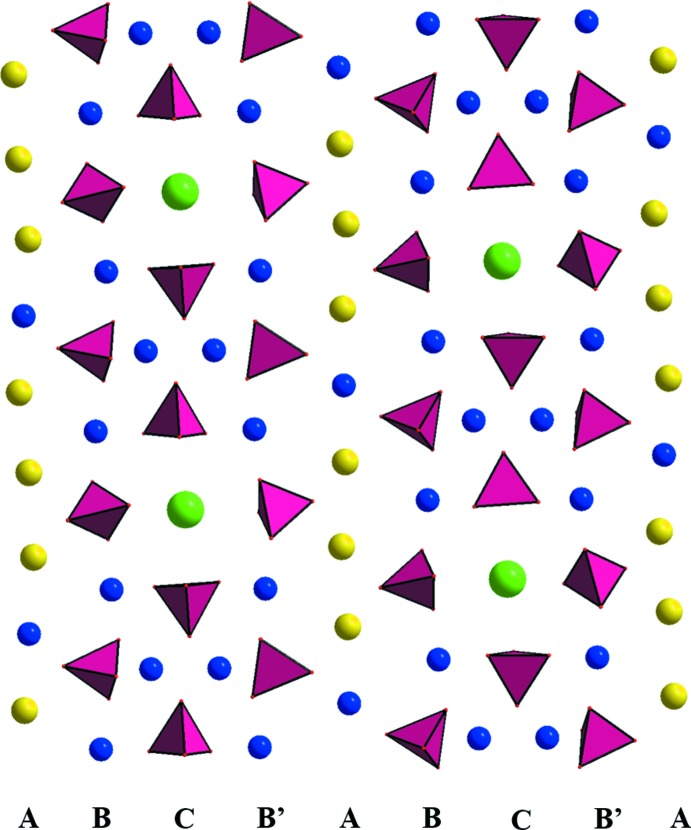
A view down the *b* axis, showing *ABCB*’ rows made of PO_4_ tetra­hedra and Mg, Na and Rb atoms.

**Figure 3:**
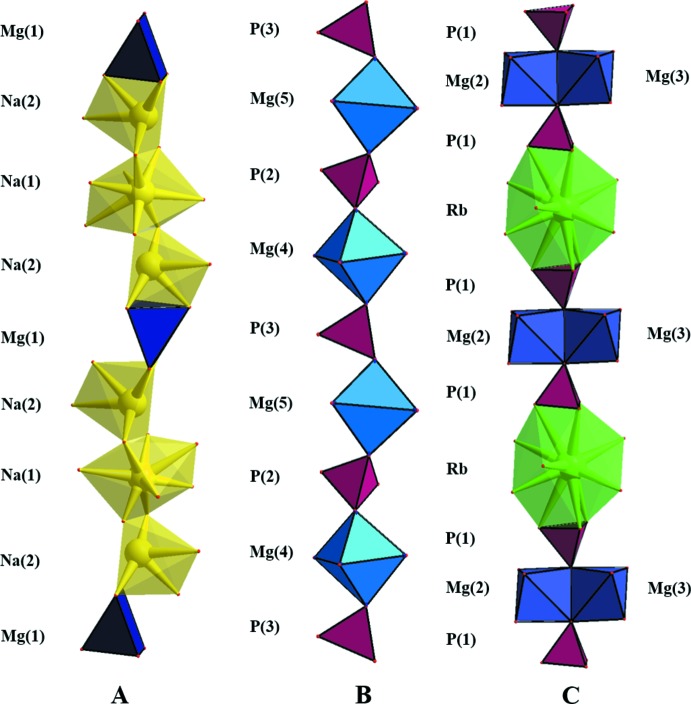
A view of parallel rows of *ABC* polyhedra.

A projection of the structure on the (012) plane (Fig. 2 ▸) shows that it can also be described on the basis of three kinds of rows (*A*, *B* and *C*) running parallel to the [100] direction. The first row (*A;* Fig. 3 ▸), consists of units with edge-sharing between one Na1O_8_ and two Na2O_6_ polyhedra. Such units alternate with Mg1O_5_ polyhedra, leading to the sequence –Mg1–Na2–Na1–Na2–. The second row (*B*) consists of corner-sharing P2O_4_, P3O_4_, Mg4O_6_ and Mg5O_5_ polyhedra, forming the sequence –P3–Mg4–P2–Mg5–. Rows *B* and *B*′ are symmetrical with respect to the inversion centre located on the *A* row. The last row (*C*) includes units with corner-sharing between P1O_4_ tetra­hedra and Mg_2_O_10_ dimers, which consist of edge-sharing Mg*i*O_6_ (*i* = 2, 3) octa­hedra. These units alternate with RbO_12_ polyhedra to form a –P1–[Mg2,Mg3]–P1–Rb– sequence. These rows, connected to each other through common corners or edges, occur with a sequence of *ABCB*′.

There are five distinct Mg sites. The Mg1 atom is displaced slightly from the inversion center, statistically occupying two symmetry-related positions. As a consequence, the Mg1O_6_ polyhedron exhibits two distances that are long [2.241 (5) Å] compared to the other Mg1—O distances, which vary from 1.969 (10) to 2.030 (10) Å. Thus, this environment can be considered as [4 + 1]. The average value of 2.005 (10) Å calculated from the four short distances is slightly higher but consistent with that of 1.930 (2) Å reported for the tetra-coordinated Mg^2+^ cation in KMgPO_4_ (Wallez *et al.*,1998 ▸). Sites Mg2 and Mg3 are located on twofold rotation axes and have slightly distorted octa­hedral environments with Mg—O distances varying from 2.052 (3) to 2.202 (2) Å for Mg2 and from 2.042 (2) to 2.169 (2) Å for Mg3. The corresponding average values of 2.123 and 2.103 Å, respectively, are in a good agreement with that of 2.14 Å observed for hexa-coordinated Mg^2+^ ions in Mg_3_(PO_4_)_2_ (Jaulmes *et al.*, 1997 ▸). Site Mg4 is [5 + 1]-coordinated, with five short distances varying from 1.981 (3) to 2.050 (3) Å and a sixth longer distance of 2.5734 (3) Å. A similar environment has already been observed in Mg_3_(PO_4_)_2_ (Jaulmes *et al.*, 1997 ▸). Site Mg5 is five-coordinated with Mg—O distances ranging from 2.020 (3) to 2.148 (3) Å. The corresponding mean distance of 2.07 Å is close to that of 2.08 Å observed for Mg^2+^ with the same coordination in NaMg_4_(PO_4_)_3_ (Ben Amara *et al.*, 1983 ▸). The P—O distances within the PO_4_ tetra­hedra are in the range of 1.518 (2)–1.552 (2) Å with an overall mean value of 1.539 Å, very close to that of 1.537 Å predicted by Baur (1974 ▸) for monophosphate groups.

**Figure 4:**
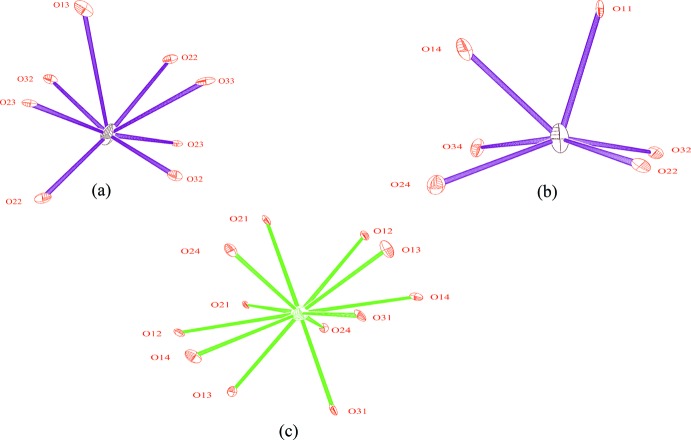
The environment of the (*a*) Na1^+^, (*b*) Na2^+^ and (*c*) Rb^+^ cations, showing displacement ellipsoids drawn at the 50% probability level.

The environments of the alkali cations are shown in Fig. 4 ▸. Those of the two crystallographic distinct Na sites were determined assuming a maximum sodium–oxygen distance *L*_max_ of 3.13 Å, as suggested by Donnay & Allmann (1970 ▸). As in the case of the Mg1 atom, the sodium atom Na1 is also moved slightly away from the inversion center and statistically occupying two symmetry-related positions. This moving probably occurs to accommodate the environment of the Na1 site, which then consists of eight oxygen atoms with Na—O distances varying from 2.303 (7) to 2.963 (6) Å. Na2 is bound to only six oxygen atoms, with Na2—O distances in the range 2.246 (3)–2.962 (3) Å. The Rb^+^ ion is located on a twofold rotation axis and occupies a single site whose environment was determined assuming all Rb—O distances to be shorter than the shortest distance between Rb^+^ and its nearest cation. This environment then consists of twelve oxygen atoms with Rb—O distances ranging from 2.923 (3) to 3.517 (2) Å.

## Synthesis and crystallization   

Single crystals of Na_3_RbMg_7_(PO_4_)_6_ were grown in a flux of sodium molybdate, Na_2_MoO_4_, with a P:Mo atomic ratio of 2:1. Appropriate amounts of the starting reactants (NH_4_)H_2_PO_4_, Na_2_CO_3_, Rb_2_CO_3_, (MgCO_3_)_4_Mg(OH)_2_·5H_2_O and Na_2_MoO_4_·2H_2_O were dissolved in nitric acid and the obtained solution was evaporated to dryness. The residue was homogenized by grinding in an agate mortar, and subsequently heated in a platinum crucible for 24 h at 673 K and then for 12 h at 873 K. After being reground, the sample was melted for 2 h at 1273 K and then cooled slowly down to room temperature at a rate of 10 K h^−1^. The solidified melt was washed with boiling water to dissolve the flux. Colourless, irregularly shaped crystals were extracted from the final product.

## Refinement   

Crystal data, data collection and structure refinement details are summarized in Table 1 ▸. The refinement was performed on the basis of electric neutrality and similar works. The atomic positions are determined by comparison with the refinements reported by Jerbi *et al.* (2010*a* ▸) and McCoy *et al.* (1994 ▸).

## Supplementary Material

Crystal structure: contains datablock(s) global, I. DOI: 10.1107/S2056989017006363/br2264sup1.cif

Structure factors: contains datablock(s) I. DOI: 10.1107/S2056989017006363/br2264Isup2.hkl

CCDC reference: 1546558

Additional supporting information:  crystallographic information; 3D view; checkCIF report

## Figures and Tables

**Table 1:** Experimental details

Crystal data	
Chemical formula	Na_3_RbMg_7_(PO_4_)_6_
*M*_r_	894.43
Crystal system, space group	Monoclinic, *C*2/*c*
Temperature (K)	293
*a*, *b*, *c* (Å)	12.734 (3), 10.685 (3), 15.498 (5)
β (°)	112.83 (2)
*V* (Å^3^)	1943.5 (10)
*Z*	4
Radiation type	Mo *K*α
μ (mm^−1^)	3.47
Crystal size (mm)	0.16 × 0.10 × 0.07

Data collection	
Diffractometer	Enraf–Nonius Turbo CAD-4
Absorption correction	Part of the refinement model (Δ*F*) (Parkin *et al.*, 1995 ▸)
*T*_min_, *T*_max_	0.377, 0.485
No. of measured, independent and observed [*I* > 2σ(*I*)] reflections	2333, 2333, 1968
*R*_int_	0.020
(sin θ/λ)_max_ (Å^−1^)	0.660

Refinement	
*R*[*F* ^2^ > 2σ(*F* ^2^)], *wR*(*F* ^2^), *S*	0.036, 0.100, 1.07
No. of reflections	2333
No. of parameters	196
Δρ_max_, Δρ_min_ (e Å^−3^)	0.87, −1.49

Computer programs: *CAD-4 EXPRESS* (Enraf–Nonius, 1994 ▸), *XCAD4* (Harms & Wocadlo, 1995 ▸), *SIR92* (Altomare *et al.*, 1993 ▸), *SHELXL2014* (Sheldrick, 2015 ▸), *DIAMOND* (Brandenburg *et al.*, 1999 ▸) and *WinGX* (Farrugia, 2012 ▸).

## supplementary crystallographic information

### Crystal data

**Table:**

Mg_7_Na_3_O_24_P_6_Rb	*F*(000) = 1744
*M**_r_* = 894.43	*D*_x_ = 3.057 Mg m^−^^3^
Monoclinic, *C*2/*c*	Mo *K*α radiation, λ = 0.71073 Å
*a* = 12.734 (3) Å	Cell parameters from 25 reflections
*b* = 10.685 (3) Å	θ = 7.5–10.8°
*c* = 15.498 (5) Å	µ = 3.47 mm^−^^1^
β = 112.83 (2)°	*T* = 293 K
*V* = 1943.5 (10) Å^3^	Prism, colourless
*Z* = 4	0.16 × 0.10 × 0.07 mm

### Data collection

**Table:**

Enraf–Nonius Turbo CAD-4 diffractometer	*R*_int_ = 0.020
non–profiled ω/2τ scans	θ_max_ = 28.0°, θ_min_ = 2.6°
Absorption correction: part of the refinement model (Δ*F*) (Parkin *et al.*, 1995)	*h* = −16→15
*T*_min_ = 0.377, *T*_max_ = 0.485	*k* = 0→14
2333 measured reflections	*l* = 0→20
2333 independent reflections	2 standard reflections every 60 min
1968 reflections with *I* > 2σ(*I*)	intensity decay: −2%

### Refinement

**Table:**

Refinement on *F*^2^	196 parameters
Least-squares matrix: full	0 restraints
*R*[*F*^2^ > 2σ(*F*^2^)] = 0.036	*w* = 1/[σ^2^(*F*_o_^2^) + (0.0549*P*)^2^ + 7.6361*P*] where *P* = (*F*_o_^2^ + 2*F*_c_^2^)/3
*wR*(*F*^2^) = 0.100	(Δ/σ)_max_ < 0.001
*S* = 1.07	Δρ_max_ = 0.87 e Å^−^^3^
2333 reflections	Δρ_min_ = −1.49 e Å^−^^3^

### Special details

**Table:**

Geometry. All esds (except the esd in the dihedral angle between two l.s. planes) are estimated using the full covariance matrix. The cell esds are taken into account individually in the estimation of esds in distances, angles and torsion angles; correlations between esds in cell parameters are only used when they are defined by crystal symmetry. An approximate (isotropic) treatment of cell esds is used for estimating esds involving l.s. planes.

### Fractional atomic coordinates and isotropic or equivalent isotropic displacement parameters (Å^2^)

**Table:**

	*x*	*y*	*z*	*U*_iso_*/*U*_eq_	Occ. (<1)
Rb	0.5000	0.74607 (5)	0.7500	0.02406 (16)	
Na1	0.2552 (5)	0.7550 (6)	0.0226 (3)	0.0279 (12)	0.5
Na2	−0.01858 (14)	0.76242 (14)	0.98277 (13)	0.0226 (4)	
Mg1	0.2487 (8)	0.2471 (9)	0.0142 (4)	0.0098 (10)	0.5
Mg2	0.0000	0.58859 (15)	0.7500	0.0071 (3)	
Mg3	0.5000	0.62142 (14)	0.2500	0.0059 (3)	
Mg4	0.18422 (9)	0.54259 (10)	0.12717 (7)	0.0065 (2)	
Mg5	0.19042 (9)	0.99696 (11)	0.14183 (7)	0.0081 (2)	
P1	0.28980 (7)	0.76938 (7)	0.27439 (6)	0.00668 (18)	
O11	0.41297 (19)	0.7658 (2)	0.27801 (17)	0.0097 (5)	
O12	0.25429 (18)	0.9074 (2)	0.27435 (15)	0.0084 (4)	
O13	0.2043 (2)	0.7106 (3)	0.18543 (18)	0.0164 (5)	
O14	0.2884 (2)	0.6963 (2)	0.36018 (17)	0.0146 (5)	
P2	0.41165 (6)	0.50005 (7)	0.08098 (5)	0.00531 (18)	
O21	0.53692 (18)	0.5150 (2)	0.15135 (15)	0.0084 (4)	
O22	0.38853 (19)	0.5698 (2)	0.98959 (16)	0.0113 (5)	
O23	0.34627 (19)	0.5654 (2)	0.13363 (15)	0.0085 (4)	
O24	0.38345 (19)	0.3619 (2)	0.06370 (17)	0.0119 (5)	
P3	0.09357 (6)	0.50198 (7)	0.92791 (5)	0.00484 (18)	
O31	−0.03126 (18)	0.4851 (2)	0.86059 (16)	0.0100 (5)	
O32	0.10990 (18)	0.6126 (2)	0.99444 (15)	0.0103 (5)	
O33	0.15211 (18)	0.5301 (2)	0.85956 (16)	0.0109 (5)	
O34	0.1418 (2)	0.3884 (2)	0.99025 (18)	0.0136 (5)	

### Atomic displacement parameters (Å^2^)

**Table:**

	*U*^11^	*U*^22^	*U*^33^	*U*^12^	*U*^13^	*U*^23^
Rb	0.0278 (3)	0.0250 (3)	0.0200 (3)	0.000	0.0099 (2)	0.000
Na1	0.0132 (16)	0.0218 (17)	0.050 (4)	−0.0034 (12)	0.014 (3)	0.009 (3)
Na2	0.0180 (8)	0.0166 (7)	0.0412 (10)	−0.0006 (6)	0.0202 (7)	−0.0027 (6)
Mg1	0.0043 (9)	0.0081 (11)	0.015 (3)	0.0002 (8)	0.001 (2)	−0.001 (3)
Mg2	0.0052 (7)	0.0109 (7)	0.0054 (7)	0.000	0.0021 (5)	0.000
Mg3	0.0044 (7)	0.0099 (7)	0.0050 (6)	0.000	0.0037 (5)	0.000
Mg4	0.0035 (5)	0.0114 (5)	0.0052 (5)	−0.0010 (4)	0.0024 (4)	0.0014 (4)
Mg5	0.0036 (5)	0.0166 (6)	0.0055 (5)	0.0009 (4)	0.0033 (4)	−0.0002 (4)
P1	0.0030 (3)	0.0093 (4)	0.0098 (4)	−0.0003 (3)	0.0046 (3)	−0.0010 (3)
O11	0.0045 (10)	0.0115 (11)	0.0158 (11)	0.0007 (8)	0.0070 (9)	0.0000 (9)
O12	0.0059 (10)	0.0106 (11)	0.0097 (11)	0.0019 (8)	0.0042 (9)	−0.0004 (8)
O13	0.0076 (11)	0.0216 (13)	0.0179 (12)	−0.0027 (10)	0.0026 (9)	−0.0117 (11)
O14	0.0149 (12)	0.0142 (12)	0.0203 (13)	0.0005 (10)	0.0129 (10)	0.0051 (10)
P2	0.0029 (4)	0.0105 (4)	0.0038 (4)	−0.0006 (3)	0.0028 (3)	−0.0011 (3)
O21	0.0027 (10)	0.0169 (11)	0.0056 (10)	−0.0002 (8)	0.0017 (8)	−0.0028 (8)
O22	0.0098 (11)	0.0191 (12)	0.0060 (10)	−0.0007 (9)	0.0043 (9)	0.0011 (9)
O23	0.0065 (10)	0.0139 (11)	0.0072 (10)	0.0000 (8)	0.0049 (8)	−0.0026 (9)
O24	0.0081 (11)	0.0112 (11)	0.0165 (12)	−0.0023 (9)	0.0049 (9)	−0.0042 (9)
P3	0.0025 (4)	0.0090 (4)	0.0040 (4)	0.0005 (3)	0.0024 (3)	0.0008 (3)
O31	0.0027 (10)	0.0181 (12)	0.0089 (11)	−0.0016 (9)	0.0018 (8)	−0.0003 (9)
O32	0.0091 (11)	0.0144 (11)	0.0082 (11)	0.0003 (9)	0.0041 (9)	−0.0034 (9)
O33	0.0044 (10)	0.0232 (12)	0.0066 (10)	0.0001 (9)	0.0037 (8)	0.0014 (9)
O34	0.0093 (11)	0.0141 (11)	0.0197 (12)	0.0061 (9)	0.0080 (9)	0.0088 (10)

### Geometric parameters (Å, º)

**Table:**

Rb—O24^i^	2.923 (3)	Mg2—O33^xvi^	2.115 (2)
Rb—O24^ii^	2.923 (3)	Mg2—O33	2.115 (2)
Rb—O13^iii^	3.163 (3)	Mg2—O31	2.202 (2)
Rb—O13^iv^	3.163 (3)	Mg2—O31^xvi^	2.202 (2)
Rb—O31^v^	3.186 (2)	Mg3—O11	2.042 (2)
Rb—O31^vi^	3.186 (2)	Mg3—O11^xvii^	2.042 (2)
Rb—O21^i^	3.301 (2)	Mg3—O21	2.100 (2)
Rb—O21^ii^	3.301 (2)	Mg3—O21^xvii^	2.100 (2)
Rb—O14^iii^	3.452 (3)	Mg3—O23^xvii^	2.169 (2)
Rb—O14^iv^	3.452 (3)	Mg3—O23	2.169 (2)
Rb—O12^iv^	3.517 (2)	Mg4—O13	1.981 (3)
Rb—O12^iii^	3.517 (2)	Mg4—O12^xv^	2.026 (3)
Na1—O32^vii^	2.303 (7)	Mg4—O23	2.041 (2)
Na1—O32^iv^	2.318 (7)	Mg4—O32^vii^	2.043 (3)
Na1—O22^iv^	2.573 (7)	Mg4—O31^xviii^	2.050 (2)
Na1—O23	2.620 (7)	Mg4—O34^vii^	2.573 (3)
Na1—O22^vii^	2.782 (7)	Mg5—O22^iv^	2.020 (3)
Na1—O13	2.878 (5)	Mg5—O21^xix^	2.026 (2)
Na1—O33^iv^	2.886 (6)	Mg5—O33^iv^	2.034 (2)
Na1—O23^viii^	2.963 (6)	Mg5—O12	2.121 (3)
Na2—O32	2.246 (3)	Mg5—O14^xx^	2.148 (3)
Na2—O24^ix^	2.340 (3)	P1—O13	1.521 (3)
Na2—O22^x^	2.365 (3)	P1—O12	1.542 (2)
Na2—O34^xi^	2.396 (3)	P1—O11	1.548 (2)
Na2—O14^xii^	2.495 (3)	P1—O14	1.548 (2)
Na2—O11^xii^	2.962 (3)	P2—O24	1.518 (2)
Mg1—O34^vii^	1.969 (10)	P2—O22^vii^	1.524 (2)
Mg1—O24	2.004 (10)	P2—O23	1.541 (2)
Mg1—O24^xiii^	2.017 (10)	P2—O21	1.552 (2)
Mg1—O34^xiv^	2.030 (10)	P3—O34	1.524 (2)
Mg1—O14^xv^	2.241 (4)	P3—O32	1.528 (2)
Mg2—O11^iv^	2.052 (3)	P3—O31	1.537 (2)
Mg2—O11^xii^	2.052 (3)	P3—O33	1.543 (2)
			
O24^i^—Rb—O24^ii^	133.51 (9)	O24^ix^—Na2—O22^x^	92.29 (10)
O24^i^—Rb—O13^iii^	84.83 (7)	O32—Na2—O34^xi^	90.71 (10)
O24^ii^—Rb—O13^iii^	101.86 (7)	O24^ix^—Na2—O34^xi^	71.97 (10)
O24^i^—Rb—O13^iv^	101.86 (7)	O22^x^—Na2—O34^xi^	159.77 (12)
O24^ii^—Rb—O13^iv^	84.83 (7)	O32—Na2—O14^xii^	131.25 (11)
O13^iii^—Rb—O13^iv^	163.19 (10)	O24^ix^—Na2—O14^xii^	75.82 (9)
O24^i^—Rb—O31^v^	136.57 (6)	O22^x^—Na2—O14^xii^	114.69 (10)
O24^ii^—Rb—O31^v^	84.63 (6)	O34^xi^—Na2—O14^xii^	74.54 (9)
O13^iii^—Rb—O31^v^	110.05 (7)	O32—Na2—O11^xii^	85.19 (9)
O13^iv^—Rb—O31^v^	54.74 (6)	O24^ix^—Na2—O11^xii^	128.61 (10)
O24^i^—Rb—O31^vi^	84.63 (6)	O22^x^—Na2—O11^xii^	99.52 (9)
O24^ii^—Rb—O31^vi^	136.57 (6)	O34^xi^—Na2—O11^xii^	100.27 (10)
O13^iii^—Rb—O31^vi^	54.74 (6)	O14^xii^—Na2—O11^xii^	53.75 (8)
O13^iv^—Rb—O31^vi^	110.05 (7)	O34^vii^—Mg1—O24	91.7 (4)
O31^v^—Rb—O31^vi^	73.42 (9)	O34^vii^—Mg1—O24^xiii^	88.6 (4)
O24^i^—Rb—O21^i^	47.00 (6)	O24—Mg1—O24^xiii^	166.9 (2)
O24^ii^—Rb—O21^i^	90.86 (6)	O34^vii^—Mg1—O34^xiv^	167.0 (2)
O13^iii^—Rb—O21^i^	72.22 (6)	O24—Mg1—O34^xiv^	87.2 (4)
O13^iv^—Rb—O21^i^	123.54 (6)	O24^xiii^—Mg1—O34^xiv^	89.6 (4)
O31^v^—Rb—O21^i^	175.28 (6)	O34^vii^—Mg1—O14^xv^	89.2 (3)
O31^vi^—Rb—O21^i^	110.99 (6)	O24—Mg1—O14^xv^	104.7 (3)
O24^i^—Rb—O21^ii^	90.86 (6)	O24^xiii^—Mg1—O14^xv^	88.4 (3)
O24^ii^—Rb—O21^ii^	47.00 (6)	O34^xiv^—Mg1—O14^xv^	103.6 (3)
O13^iii^—Rb—O21^ii^	123.54 (6)	O11^iv^—Mg2—O11^xii^	81.37 (14)
O13^iv^—Rb—O21^ii^	72.22 (7)	O11^iv^—Mg2—O33^xvi^	117.11 (10)
O31^v^—Rb—O21^ii^	110.99 (6)	O11^xii^—Mg2—O33^xvi^	89.56 (10)
O31^vi^—Rb—O21^ii^	175.28 (6)	O11^iv^—Mg2—O33	89.56 (10)
O21^i^—Rb—O21^ii^	64.66 (8)	O11^xii^—Mg2—O33	117.11 (10)
O24^i^—Rb—O14^iii^	126.45 (6)	O33^xvi^—Mg2—O33	145.64 (16)
O24^ii^—Rb—O14^iii^	63.05 (6)	O11^iv^—Mg2—O31	145.19 (10)
O13^iii^—Rb—O14^iii^	44.16 (6)	O11^xii^—Mg2—O31	86.60 (9)
O13^iv^—Rb—O14^iii^	131.70 (6)	O33^xvi^—Mg2—O31	95.21 (10)
O31^v^—Rb—O14^iii^	85.51 (6)	O33—Mg2—O31	67.21 (9)
O31^vi^—Rb—O14^iii^	78.00 (6)	O11^iv^—Mg2—O31^xvi^	86.60 (9)
O21^i^—Rb—O14^iii^	93.70 (6)	O11^xii^—Mg2—O31^xvi^	145.19 (10)
O21^ii^—Rb—O14^iii^	103.71 (6)	O33^xvi^—Mg2—O31^xvi^	67.21 (9)
O24^i^—Rb—O14^iv^	63.05 (6)	O33—Mg2—O31^xvi^	95.20 (9)
O24^ii^—Rb—O14^iv^	126.45 (6)	O31—Mg2—O31^xvi^	119.70 (14)
O13^iii^—Rb—O14^iv^	131.70 (6)	O11—Mg3—O11^xvii^	81.88 (14)
O13^iv^—Rb—O14^iv^	44.16 (6)	O11—Mg3—O21	149.00 (10)
O31^v^—Rb—O14^iv^	78.00 (6)	O11^xvii^—Mg3—O21	87.76 (9)
O31^vi^—Rb—O14^iv^	85.51 (6)	O11—Mg3—O21^xvii^	87.76 (9)
O21^i^—Rb—O14^iv^	103.71 (6)	O11^xvii^—Mg3—O21^xvii^	149.00 (10)
O21^ii^—Rb—O14^iv^	93.70 (6)	O21—Mg3—O21^xvii^	114.43 (14)
O14^iii^—Rb—O14^iv^	159.44 (9)	O11—Mg3—O23^xvii^	114.89 (10)
O24^i^—Rb—O12^iv^	67.45 (6)	O11^xvii^—Mg3—O23^xvii^	89.77 (9)
O24^ii^—Rb—O12^iv^	90.89 (6)	O21—Mg3—O23^xvii^	94.09 (9)
O13^iii^—Rb—O12^iv^	150.50 (6)	O21^xvii^—Mg3—O23^xvii^	68.28 (9)
O13^iv^—Rb—O12^iv^	42.77 (6)	O11—Mg3—O23	89.77 (9)
O31^v^—Rb—O12^iv^	97.43 (6)	O11^xvii^—Mg3—O23	114.89 (10)
O31^vi^—Rb—O12^iv^	128.18 (6)	O21—Mg3—O23	68.28 (9)
O21^i^—Rb—O12^iv^	81.20 (5)	O21^xvii^—Mg3—O23	94.09 (9)
O21^ii^—Rb—O12^iv^	50.59 (5)	O23^xvii^—Mg3—O23	147.97 (14)
O14^iii^—Rb—O12^iv^	153.49 (6)	O13—Mg4—O12^xv^	111.06 (11)
O14^iv^—Rb—O12^iv^	43.24 (6)	O13—Mg4—O23	85.41 (10)
O24^i^—Rb—O12^iii^	90.89 (6)	O12^xv^—Mg4—O23	87.74 (10)
O24^ii^—Rb—O12^iii^	67.45 (6)	O13—Mg4—O32^vii^	93.13 (12)
O13^iii^—Rb—O12^iii^	42.77 (6)	O12^xv^—Mg4—O32^vii^	155.80 (11)
O13^iv^—Rb—O12^iii^	150.50 (6)	O23—Mg4—O32^vii^	94.02 (10)
O31^v^—Rb—O12^iii^	128.18 (6)	O13—Mg4—O31^xviii^	92.75 (11)
O31^vi^—Rb—O12^iii^	97.43 (6)	O12^xv^—Mg4—O31^xviii^	86.03 (10)
O21^i^—Rb—O12^iii^	50.59 (5)	O23—Mg4—O31^xviii^	172.38 (11)
O21^ii^—Rb—O12^iii^	81.20 (5)	O32^vii^—Mg4—O31^xviii^	93.46 (10)
O14^iii^—Rb—O12^iii^	43.24 (6)	O13—Mg4—O34^vii^	154.80 (11)
O14^iv^—Rb—O12^iii^	153.49 (6)	O12^xv^—Mg4—O34^vii^	93.48 (10)
O12^iv^—Rb—O12^iii^	124.43 (8)	O23—Mg4—O34^vii^	90.10 (9)
O32^vii^—Na1—O32^iv^	163.38 (18)	O32^vii^—Mg4—O34^vii^	62.42 (9)
O32^vii^—Na1—O22^iv^	88.3 (2)	O31^xviii^—Mg4—O34^vii^	94.64 (9)
O32^iv^—Na1—O22^iv^	94.9 (3)	O22^iv^—Mg5—O21^xix^	89.44 (10)
O32^vii^—Na1—O23	74.4 (2)	O22^iv^—Mg5—O33^iv^	92.64 (10)
O32^iv^—Na1—O23	112.8 (3)	O21^xix^—Mg5—O33^iv^	175.75 (11)
O22^iv^—Na1—O23	137.03 (19)	O22^iv^—Mg5—O12	132.14 (11)
O32^vii^—Na1—O22^vii^	89.9 (2)	O21^xix^—Mg5—O12	89.48 (10)
O32^iv^—Na1—O22^vii^	83.2 (2)	O33^iv^—Mg5—O12	86.38 (10)
O22^iv^—Na1—O22^vii^	166.37 (17)	O22^iv^—Mg5—O14^xx^	110.57 (11)
O23—Na1—O22^vii^	54.84 (15)	O21^xix^—Mg5—O14^xx^	92.12 (10)
Na1^viii^—Na1—O13	150.6 (12)	O33^iv^—Mg5—O14^xx^	90.63 (10)
O32^vii^—Na1—O13	67.64 (15)	O12—Mg5—O14^xx^	117.28 (10)
O32^iv^—Na1—O13	129.0 (2)	O13—P1—O12	106.71 (14)
O22^iv^—Na1—O13	77.79 (16)	O13—P1—O11	112.40 (14)
O23—Na1—O13	59.29 (12)	O12—P1—O11	108.47 (12)
O22^vii^—Na1—O13	113.9 (2)	O13—P1—O14	109.11 (15)
O32^vii^—Na1—O33^iv^	138.3 (2)	O12—P1—O14	112.44 (13)
O32^iv^—Na1—O33^iv^	56.44 (15)	O11—P1—O14	107.79 (13)
O22^iv^—Na1—O33^iv^	64.67 (16)	O24—P2—O22^vii^	111.34 (14)
O23—Na1—O33^iv^	103.37 (17)	O24—P2—O23	113.25 (13)
O22^vii^—Na1—O33^iv^	123.6 (2)	O22^vii^—P2—O23	108.74 (13)
O13—Na1—O33^iv^	75.63 (12)	O24—P2—O21	109.41 (13)
O32^vii^—Na1—O23^viii^	102.1 (2)	O22^vii^—P2—O21	112.20 (13)
O32^iv^—Na1—O23^viii^	67.63 (16)	O23—P2—O21	101.56 (12)
O22^iv^—Na1—O23^viii^	52.91 (14)	O34—P3—O32	105.79 (14)
O23—Na1—O23^viii^	168.2 (2)	O34—P3—O31	113.15 (14)
O22^vii^—Na1—O23^viii^	114.48 (15)	O32—P3—O31	112.52 (13)
O13—Na1—O23^viii^	130.4 (2)	O34—P3—O33	114.02 (13)
O33^iv^—Na1—O23^viii^	86.8 (2)	O32—P3—O33	109.69 (14)
O32—Na2—O24^ix^	143.52 (12)	O31—P3—O33	101.82 (13)
O32—Na2—O22^x^	95.11 (10)		

Symmetry codes: (i) *x*, −*y*+1, *z*+1/2; (ii) −*x*+1, −*y*+1, −*z*+1; (iii) *x*+1/2, −*y*+3/2, *z*+1/2; (iv) −*x*+1/2, −*y*+3/2, −*z*+1; (v) *x*+1/2, *y*+1/2, *z*; (vi) −*x*+1/2, *y*+1/2, −*z*+3/2; (vii) *x*, *y*, *z*−1; (viii) −*x*+1/2, −*y*+3/2, −*z*; (ix) *x*−1/2, *y*+1/2, *z*+1; (x) −*x*+1/2, −*y*+3/2, −*z*+2; (xi) −*x*, −*y*+1, −*z*+2; (xii) *x*−1/2, −*y*+3/2, *z*+1/2; (xiii) −*x*+1/2, −*y*+1/2, −*z*; (xiv) −*x*+1/2, −*y*+1/2, −*z*+1; (xv) −*x*+1/2, *y*−1/2, −*z*+1/2; (xvi) −*x*, *y*, −*z*+3/2; (xvii) −*x*+1, *y*, −*z*+1/2; (xviii) −*x*, −*y*+1, −*z*+1; (xix) *x*−1/2, *y*+1/2, *z*; (xx) −*x*+1/2, *y*+1/2, −*z*+1/2.
